# Evidence of the Association between Nurse Staffing Levels and Patient and Nurses’ Outcomes in Acute Care Hospitals across Japan: A Scoping Review

**DOI:** 10.3390/healthcare10061052

**Published:** 2022-06-06

**Authors:** Noriko Morioka, Suguru Okubo, Mutsuko Moriwaki, Kenshi Hayashida

**Affiliations:** 1 Graduate School of Health Care Sciences, Tokyo Medical and Dental University, Tokyo 113-8519, Japan; 2 Institute of Ars Vivendi, Ritsumeikan University, Kyoto 603-8577, Japan; suguruokubo@gmail.com; 3 Quality Management Center Medical Hospital, Tokyo Medical and Dental University, Tokyo 113-8519, Japan; mmoriwaki.qmc@tmd.ac.jp; 4 Department of Medical Informatics and Management, University Hospital, University of Occupational and Environmental Health, Fukuoka 807-8556, Japan; kenshi@clnc.uoeh-u.ac.jp

**Keywords:** nurse staffing ratio, patient safety, nurse sensitivity outcome, nurse sensitivity indicator, quality of nursing

## Abstract

We aimed to summarize the evidence of an association between nurse staffing and nursing sensitivity outcomes in Japanese hospitals. A scoping review was conducted and reported following the PRISMA-SR 2020 statement. The ICHUSHI and CiNii databases were searched for published articles written in Japanese and PubMed and CINAHL for those written in English. Out of the 15 included studies, all observational studies, 3 were written in Japanese and the others in English. The nurse staffing level measures were grouped into three categories: patient-to-nurse ratio, nursing hours per patient day, and nurse-to-bed ratio. The outcome measures were grouped into three categories: patient outcome, nursing care quality reported by nurses, and nurse outcome/nursing care quality. Some studies reported that the nursing staff increasingly favored positive patient outcome. Conversely, the findings regarding failure to rescue, in-hospital fracture, and post-operative complications were inconsistent. Although some studies indicated that more nurse staffing was favored toward better patient and nurse outcomes, due to the sparse accumulation of studies and heterogeneity among the findings, it is difficult to draw robust conclusions between nurse staffing level and outcomes in Japanese acute care hospitals.

## 1. Introduction

Globally, nurses are the largest component of the healthcare profession. To deliver qualitative care and patient safety, assigning an effective nursing workforce and ensuring evidence-based nurse staffing have been important issues in health policy. Following the monumental works of Aiken [1] and Needleman [2], numerous studies have investigated the association between nurse staffing and nurse sensitive outcomes for more than three decades. Recently, an umbrella review summarized the vast existing evidence between nurse staffing and patient outcomes [3]. However, the causal inference and complex findings are still not clear based on the majority of studies from the U.S. and European countries with a cross-sectional design.

Japan has also faced challenges in ensuring adequate nursing staff to deliver quality care. It employed a unique regulation for minimum nurse staffing ratio as a combination of law and financial scheme in the National Health Insurance. First, the minimum ratio of the total number of patients to the total number of employed nurses (full-time equivalent number, FTE) was regulated in the Medical Care Act; 3:1 for inpatient and 30:1 for outpatient, i.e., a hospital with 500 inpatients and 3000 outpatients should employ 267 or more FTE nurses (500/3 + 3000/30 = 167 + 100 = 267). All hospitals are required to maintain this mandate for the minimum ratio. Second, the fee schedule in the National Health Insurance system also set nurse staffing standards for each functional category of inpatient wards [4]. The required number of nurses as per the Medical Care Act is just the minimum number, and hospitals actually employ more nurses to keep the nurse staffing level in the fee schedule. In 2006, the nurse staffing measure was revised from a patient-to-employed nurse ratio (which used the same calculation as the nurse staffing measure in the Medical Care Act) to a patient-to-nurse ratio per shift. There are currently four nurse staffing level standards in general care wards: 7:1, 10:1, 13:1, and 15:1. Hospitals should choose one category and assign more nurses than those indicated by the average daily number of inpatients per nursing staff per shift in all their general, surgical, and medical wards to gain the basic fee for inpatients. Although research has been conducted since Kanda’s report [5], larger sample studies investigating the effect of nurse staffing regulation on patient and nurse outcomes are necessary, similar to other countries [6]. No study has reviewed the comprehensive evidence on nurse staffing and nursing sensitivity outcomes in Japan. Therefore, this study aimed to identify the variables used in studies related to nurse staffing and outcomes and summarize the association between nurse staffing and nursing sensitivity outcomes in Japan. This review will provide evidence under the unique regulation for minimum nurse staffing ratio as a combination of law and financial scheme in the National Health Insurance system and contribute by comparing the findings in other countries with only law regulation or without mandated staffing ratio.

## 2. Materials and Methods

To identify and map the available evidence of an association between nurse staffing and nursing sensitivity outcomes in Japan, a scoping review was conducted. All results were reported following the Preferred Reporting Items for Systematic Reviews and Meta-Analyses for Scoping Review (PRISMA-SR) 2020 statement [7].

### 2.1. Search Strategy

We searched for published studies via the ICHUSHI and CiNii databases for those written in Japanese and PubMed and CINAHL databases for those in English. We used search terms ‘nurse and (staffing or number or deployment or ratio or rate or proportion)’, ‘quantity of nursing care’, ‘nursing system’, ‘patient and outcome’, ‘nurse sensitive outcome’, ‘nurse sensitive indicator’, ‘patient satisfaction’, ‘quality of nursing care’, ‘job satisfaction’, ‘burnout’, ‘Japan’, and ‘hospital’. The detailed search strategies are shown in Table S1. We set the lower date limit to December 2021.

### 2.2. Inclusion Criteria

We included studies that met the following criteria: (1) written in English or Japanese, (2) original article with quantitative research, (3) target population/participants were patients or nurses, (4) acute care setting/hospital in Japan, (5) included nurse staffing variables, (6) examined the association between nurse staffing and outcomes, and (7) described the association between nurse staffing and outcomes.

### 2.3. Study Selection

First, one reviewer (SO) conducted a screening of the titles and abstracts to remove duplicates and irrelevant studies. Next, two reviewers (N.M., S.O.) independently assessed the full texts as per the inclusion criteria. Any disagreements were resolved by discussion between the two reviewers. The selection processes were managed by the Rayyan systematic review software (Qatar Computing Research Institute).

### 2.4. Assessment of the Risk of Bias

For a critical appraisal of the individual sources of evidence, we used the Risk Of Bias In Non-randomized Studies of Interventions (ROBINS-I) tool [8]. The ROBINS-I tool included seven domains that were assessed: confounding, selection of the participants, classification of the interventions, deviation from the intended interventions, missing data, measurement of the outcomes, and selection of the reported results. The risk of bias for each domain was graded on four levels: low, moderate, serious, or critical. The overall risk of bias was determined by the grade of the domain with the highest risk. In the ROBINS-I tool, there were two domains regarding intervention or exposure. With reference to the methods used in a previous review [9], we interpreted ‘intervention’ as a variation in exposure to nurse staffing levels when the included study was an observational study. Two reviewers (N.M. and S.O.) independently evaluated the risk of bias of the included studies and reached consensus by discussion in case of any disagreements.

### 2.5. Data Extraction and Synthesis

From each study included, we extracted the author(s), year, design and data sources, setting, participants (including sample size), measure(s) of the nurse staffing level, measure(s) of the outcome measure(s), analysis and risk adjustment for potential confounder, and the findings. We categorized the measure(s) of nurse staffing level and outcome measure(s) into groups and summarized the findings of the association between nurse staffing level and outcomes.

## 3. Results

### 3.1. Search Results

**Figure 1:**
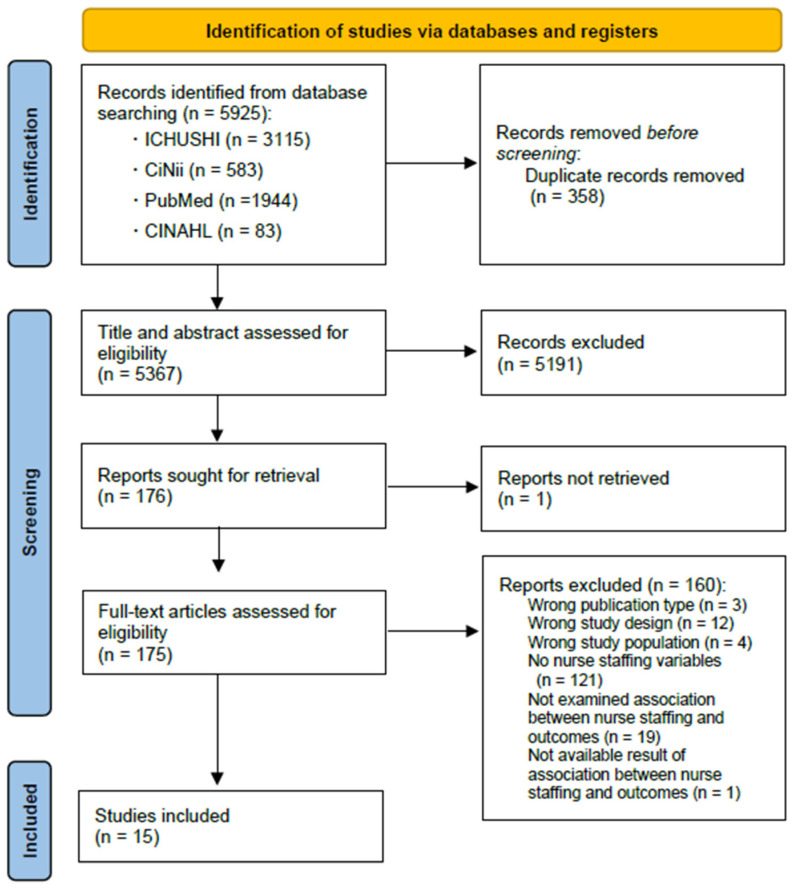
Flow chart of the study selection process.

We identified 5925 articles via the database searches. After 358 duplicates were removed, 5367 articles were identified. These were assessed for eligibility, and 15 studies met the inclusion criteria. Figure 1 details a flow diagram of the article selection process.

### 3.2. Study Characteristics

Out of the 15 studies, 3 were written in Japanese [10,11,12], and the others in English [13,14,15,16,17,18,19,20,21,22,23]. The studies were all observational in nature (Table 1 and Table S2). Nine studies used self-administered questionnaire surveys among hospital nurses or patients, and of those, four employed a two-wave [14,21] or longitudinal design [17,24] and five a cross-sectional design [10,11,12,13,22]. The number of participants in each study ranged from 14 [14] to 2386 [21].

Six studies were large-sample retrospective observational studies using clinical databases, one using an original clinically registered database [15] and the others the Diagnosis Procedure Combination (DPC) database [16,18,19,20,23]. The DPC database, a nationwide database of acute care inpatients, comprised administrative claims data and discharge information on approximately 0.48 million beds in 1757 hospitals in Japan [25].

Of the nine studies where the target population were patients, five studies used surgical patients [16,18,19,20,23], two used psychiatric patients [15,21], and two had no restrictions [11,17].

Seven studies did not include the confounders in the analysis, which indicated that the findings were not risk adjusted [10,11,12,13,14,21,22,24]. The overall risk of bias assessment judgments of the 15 studies varied from moderate to critical (Table S3).

### 3.3. Nurse Staffing Level Measures

The nurse staffing level measures in the 15 studies were grouped into three categories: (1) patient-to-nurse ratio, (2) nursing hours per patient per day, and (3) nurse-to-bed ratio (Table 1, Table 2 and Table S2). The patient-to-nurse ratio was used in 11 studies. Patient-to-nurse ratio consisted of patient-to-nurse ratio requirement in the fee schedule (7:1 group, 10:1 group) and patient-to-nurse ratio per shift. Patient-to-nurse ratio per shift was divided into two types: one, the average of all/target wards at the hospital level variable obtained from calculating the inpatient number and the number of full-time equivalent nurses (patient-to-nurse ratio per shift = (total inpatient days × 24 hours) / (number of full equivalent nurses × 1800 hours)) [26] and two, nurses’ variable at the individual level obtained through questionnaires. Nursing hours per patient day (NHPPD) was used in one study, in which the NHPPD was at each ward level. Nurse-to-bed ratio was used in three studies; two used the number of occupied beds, and the other used the total number of beds, also divided into hospital and ward levels.

### 3.4. Outcome Measures

The outcome measures in the 15 studies were grouped into three categories: (1) patient outcome, (2) nursing care quality reported by nurses, and (3) nurse outcome (Table 1, Table 2 and Table S2). Regarding patient outcome, the following outcomes were examined: readmission in one study, hospitalization in one study, in-hospital mortality in four studies, failure to rescue in two studies, lengths of hospital stay in two studies, in-hospital pneumonia in one study, in-hospital fracture in two studies, post-operative complications in two studies, seclusion in one study, pressure ulcers in one study, physical restraint in one study, error and/or near miss in one study, and patient-reported satisfaction with nursing care in one study.

Regarding nursing care quality reported by nurses, quality of care was claimed to have improved (agree to disagree) in two studies, and the ability to provide quality care (agree to disagree) was reported in one study.

Regarding nurse outcome, the following outcomes were measured: work engagement in one study, job satisfaction in one study, stressors at work in one study, response to stress in one study, intention to leave in one study, decision to leave in two studies, working environment in one study, and ward morale in one study.

### 3.5. Evidence of the Association between Nurse Staffing Level and Outcomes

Table 2 shows the summary of the evidence for the association between nurse staffing level and outcomes in Japanese hospitals. The length of hospital stay of patients aged 65 or older who underwent hip surgery was more likely to be extended, with a higher number of patient-to-nurse ratio per shift in two studies [18,19]. Pressure ulcers were less likely to occur in wards with higher NHPPD [17]. Patient satisfaction with nursing care was likely to be higher in the 7:1 patient-to-nurse group than in the 10:1 patient-to-nurse group [14]. In other words, these indicated that more nursing staff favored better patient outcome. Physical restraint was more likely used in psychiatric wards with a higher ratio of nurses to bed [15], which indicated that fewer staff favored better patient outcome. Readmission [18], hospitalization [21], in-hospital mortality [16,18,19,23], in-hospital pneumonia [19], physical restraint [17], and and/or near miss [11] were reported. However, these did not result in a statistically significant association with nurse staffing levels.

The findings regarding failure to rescue, in-hospital fracture, and post-operative complications were inconsistent. For failure to rescue, Yasunaga et al. [23] indicated that more staff (nurse and physician) per bed was associated with lower risks among patients who underwent selective cancer surgeries. For post-operative complications, Hirose et al. [16] show a reverse J-shaped association with post-operative complications, with a threshold patient-to-nurse ratio of 5.4 per shift. Hence, Hirose et al.’s study showed no statistical association with the number of nurses and physicians per bed.

For in-hospital fracture, Morita et al. [20] indicated that more nurses per occupied bed was associated with a lower risk among patients aged 50 or older who underwent major cancer and cardiovascular surgeries. Furthermore, Morioka et al.’s study [18] showed no statistical association among patients with dementia aged 65 or older who underwent hip surgery.

Both studies that investigated nursing care quality showed no statistically significant association with a patient-to-nurse ratio per shift at the individual level of nurses [13] and requirement in the fee schedule at the hospital level (7:1 group, 10:1 group) [14].

For nurses’ outcome, the studies demonstrate that more nurse staffing was associated with better job satisfaction levels [12], work environment [14], and ward morale [13]. Work engagement [10], stressors at work [14], response to stress [14], intention to leave [22], and decision to leave [22,24] were also reported and were not statistically significantly associated with nurse staffing levels. Detailed findings of the 15 studies are shown in Table S2.

## 4. Discussion

To the best of our knowledge, this is the first study to review evidence of an association between nurse staffing level and outcomes in Japanese hospitals since the introduction of the patient-to-nurse ratio per shift requirement in 2006 in Japan. Our findings were composed of all the observational studies that used questionnaire surveys or large-sample databases. We identified the measures of nurse staffing and outcomes and mapped the findings between nurse staffing and outcomes.

### 4.1. Nurse Staffing Level Measures

Patient-to-nurse ratio, NHPPD, and nurse-to-bed ratio were identified as nurse staffing measures. There were three observational levels: nurse individual level, ward (unit) level, and hospital average level. Globally, nurse staffing measures and measurement levels varied widely [3,27]. In a large international study group, Nurse Forecasting in Europe (RN4CAST), nurse staffing was measured at the hospital level as the mean number of the patients assigned to the nursing staff who reported caring for at least one patient from the nurses’ questionnaire survey [1,28]. Among the included studies, more than half used patient-to-nurse ratio per shift as the hospital-level variable according to the nurse staffing requirement category or the calculation method in the fee schedule in Japan [4,26]. This was the hospital figure that reflected the average daily number of inpatients per nursing staff per shift. The government allows for a sloping assignment of nurses among wards in a day, that is, six nurses should work every three shifts (each shift is 8 hours) in a ward with 42 inpatients under the 7:1 patient-to-nurse ratio category. However, in reality, seven or eight nurses work day shifts (5:1 or 6:1), and three nurses work night shifts (14:1). The nurse manager can assign nurses to other wards with increased workloads. Thus, the interpretation of the findings must be carried out with caution, as the actual ward (unit)-level staffing may often differ from the reported number. Regarding this problem, Spetz et al. [27] also pointed out that the unit-level data collection was preferable due to the great difference between unit-level staffing and hospital-level aggregated data. Further studies with ward-level staffing data are necessary in Japan.

### 4.2. Outcome Measures 

Patient outcome, nursing quality, and nurses’ outcome were identified as outcome measures. The identified patient outcome measures were commonly known as nursing sensitivity indicators, as defined by the National Quality Forum [29] and also in previous studies in other countries [3]. Compared with studies in other countries [3], those were just a small part of nursing sensitivity indicators. In the existing administrative database, such as the DPC database, the collection of other nurse sensitivity outcome variables might be difficult.

As for nurses’ outcome, the questionnaire survey for nurses used original items to measure their working environment and health status. Most items were based on a Likert scale and were not validated. In other countries, the validated Practice Environment Scale of the Nursing Work Index (PES-NWI) [30] was commonly used to investigate the effect on nurse staffing level on nurses’ working environment [31,32]. Hence, further studies with a validated scale for nurses’ working environment or health status are necessary in Japan.

### 4.3. Evidence of the Association between Nurse Staffing Level and Outcomes in Japanese Hospitals

Some studies indicated that more nursing staff favored a better case for the following outcomes: failure to rescue patients, length of hospital stay, post-operative complications, in-hospital fracture, pressure ulcers, patient satisfaction, nurse job satisfaction, working environment, and ward morale. The findings of some outcomes were consistent with previous studies. A meta-analysis showed that an increase by one registered nurse per patient day was associated with a lower risk of failure to rescue (OR, 0.84; 95% CI, 0.79–0.90) in surgical patients. The length of stay was shorter by 31% in surgical patients (OR, 0.69; 95% CI, 0.55–0.86) [33]. An umbrella review indicated that the strengths of evidence of nurse staffing, length of stay, quality of nurse delivery care, and readmissions were high, and failure to rescue, mortality, and pneumonia were moderate [3].

In contrast, some studies showed no significant association between nurse staffing and readmission, hospitalization, hospital mortality, pneumonia, error, nurse-reported nursing care, and intention to leave, which were inconsistent with previous studies [3,33,34]. This might be explained due to the following reasons. First, the nurse staffing variable was at hospital level in those studies. This might not reflect the actual degree of nursing care each patient received [27]. Second, the physician and medical care environment could affect patients’ adverse events. Hence, they may offset the association of nurse staffing. Third, there was a small number of studies that investigated each outcome. Further research is required to draw additional conclusions.

Importantly, the challenges in the evidence of nurse staffing level and outcome in the current studies in Japan were revealed. First, there was heterogeneity among the findings, and there were insufficient studies to examine all the variables as, except for in-hospital mortality, only one or two studies investigated each outcome. Second, most studies that investigated nurse staffing and nurse outcome did not involve a risk adjustment, which could have resulted in a risk of bias. In addition, most studies used a cross-sectional design, which made it difficult to lead to a causal inference. Studies with other designs, i.e., longitudinal or quasi-experimental studies, are necessary [34]. Third, there might be a non-linear association between nurse staffing level and outcomes. As Hirose et al. [16] indicated, non-linear association between nurse staffing level and outcomes was pointed out in a U.K. study [35]. The existing thresholds should be considered when discussing appropriate nurse staffing levels. Further research is necessary to gain robust conclusions on the association between nurse staffing levels and outcomes in Japanese hospitals. Robust evidence on nurse staffing levels and outcomes will contribute to assigning an effective nurse workforce in Japan, a country that often faces a nurse workforce shortage.

### 4.4. Limitations

This study has several limitations. First, we did not identify all the studies that investigated the association between nurse staffing and outcomes, particularly those in which nurse staffing variables were covariate in nature. Second, we used selected databases and limited languages (ones written in English or Japanese). However, these databases included most nursing studies conducted in Japan. There was little possibility that the studies conducted in Japan were written in other languages. Third, we could not include the studies at the full-text screening stage, wherein the detailed result data did not show the tables or text, even if the study included nurse staffing variables.

## 5. Conclusions

This literature review summarized the evidence of the association between nurse staffing levels and outcomes in Japanese hospitals. Some studies indicated that more nursing staff had a better outcome across the failure to rescue, length of hospital stay, post-operative complications, in-hospital fractures, pressure ulcers, nurses’ job satisfaction, working environment, and ward morale. However, some findings were inconsistent. There is a sparse accumulation of studies and heterogeneity among the findings. More robust design and wide patient characteristics are required to confirm this conclusion in future studies in Japan.

## Figures and Tables

**Table 1:** Characteristics of studies included in the systematic review.

Author	Design and Data Source	Setting, Participants, and Sample Size	Measurement of Staffing	Outcome Measures	Analysis and Potential Confounders Measured and Included in the Analysis	Main Findings
Nawata et al. [21]	Two-wave questionnaire survey (October 1993 and October 1999)	Patients (first survey) = 2386; Patients (second survey) = 1131, Psychiatric hospitals = 18	Patient-to-nurse ratio per shift (wards average at hospital level)	Percentage of followed-up period hospitalized	Bivariate analysis	No statistically significant association
Suzuki et al. [24]	Longitudinal questionnaire survey from June 2003 to December 2003	Novice full-time nurses in 2003 = 1203, University hospitals = 20	Patient-to-nurse ratio requirement in the fee schedule (hospital level) 7:1 vs. 10:1	Rapid turnover among novice nurses	Bivariate analysis	No statistically significant association
Ibe et al. [17]	Longitudinal questionnaire survey from 1 November 2005 to 31 January 2006	Hospital nursing unit managers = 98, Hospitals = 42	Registered nurse hours per patient day (ward level) Associate nurse hours per patient day (ward level)	Pressure ulcer, physical restraint	Multiple regression analysis Conf.: a patient-level variable, work environment variables	More associated nurse hours per patient (daily) related to lower number of pressure ulcer
Kaneko et al. [11]	Cross-sectional questionnaire survey during November and December 2005	Nurses = 787 (candidates = 1339), Hospitals = 6	Patient-to-nurse ratio on day/ night shift (nurse individual level)	Medical error, medical mistakes	Univariate logistic regressionConf.: age	No statistically significant association
Fujimura et al. [14]	Questionnaire survey from January 2008 to December 2008	Inpatient survey: Inpatients = 202, Hospital = 1	Patient-to-nurse ratio requirement in the fee schedule (hospital level) 7:1 vs. 10:1	Inpatient satisfaction with nursing care	Bivariate analysis	7:1 system showed statistically greater satisfaction than that in the 10:1 system
		Medical workers survey: First survey: Physicians = 36, Nurses = 14 Second survey: Physicians = 33, Nurses = 25 Hospital = 1		Job satisfaction, effects of the DPC, stress of medical workers	Bivariate analysis	Nurses under the 7:1 system judged that their working hours were shortened compared to those under the 10:1 system No statistically significant association with nurse-reported nursing care and stress
Yasunaga et al. [23]	Retrospective observational study Data: DPC, the Survey of Medical Institutions data	Patients = 131,394 (underwent elective cancer surgery between 2007 and 2008)	Number of physicians per 100 occupied beds, number of nurses per 100 occupied beds (hospital level)	Post-operative complications, in-hospital mortality, failure to rescue	Logistic regression analysis Conf.: patient-level variables, hospital volume	Higher number of patients and nurses per occupied bed associated with lower failure to rescue but not associated with post-operative complications and in-hospital mortality
Namba et al. [12]	Cross-sectional questionnaire survey from 22 April to 28 May 2009	Full-time nurses of = 919 (candidates = 2213), Hospitals = 15	Number-of-patients-to-nurse ratio (hospital level) 7:1 vs. 10:1	Job satisfaction, retention potential	Bivariate analysis	Job satisfaction in 7:1 system was higher than that in 10:1
Tei-Tominaga [22]	Cross-sectional questionnaire survey in December 2009	Newly graduated nurses = 493 (candidates = 1477), Hospitals = 353	Patient-to-nurse ratio requirement in the fee schedule (hospital level)	Intention to leave, decision to resign	Multivariate logistic regression analysis Conf.: employment and organizational characteristics, individual factors	No statistically significant association
Anzai et al. [13]	Cross-sectional questionnaire survey	Nurses = 223 (candidates = 341), Acute care inpatient wards = 12, Hospital = 1	Patient-to-nurse ratio in usual day shift (nurse individual level)	Ability to provide quality nursing care, quality of care, ward morale	Multiple regression analyses Conf.: demographic characteristics, work characteristics, work environment, management	Higher patient-to-nurse ratio in usual day shift was associated with lower ward morale but not associated with ability to provide quality nursing care
Morita et al. [20]	Retrospective cohort study Data: DPC, the Surveys for Medical Institution	Patients = 770,373 (50 years or older and underwent planned major cancer or cardiovascular surgery from July 2010 to March 2014), Hospitals = 1074	Nurse-to-occupied-bed ratio (hospital level)	The occurrence of in-hospital bone fractures	Logistic regression analysis Conf.: patient-level variables	The higher nurses-to-occupied-bed ratio was associated with lower risk of in-hospital bone fractures
Fukasawa et al. [15]	Secondary analysis of clinical database, the Psychiatric Electronic Clinical Observation (PECO) system from April 2015 to March 2017	Admissions = 10,013, Hospitals = 23	Nurse to 10 beds in each psychiatric ward (ward level)	Use of seclusion or mechanical restraint during the first 90 days of admission	Multilevel logistic regression analysis Conf.: patient-level variables, hospital(ward)-level variables	The higher number of nurses per bed was associated with higher risk of seclusion and mechanical restraint
Ito et al. [10]	Cross-sectional questionnaire survey from 5 December 2013 to 25 December 2013	Shift-work nurses = 1275 Hospitals = 13	Patient-to-nurse ratio requirement in the fee schedule (hospital level)	Work engagement	Bivariate analysis	No statistically significant association
Morioka et al. [18]	Retrospective observational study Data: DPC, reporting on medical functions of hospital beds data	Patients = 20,393 (dementia, 65 years or older, underwent hip surgeries and discharged from April 2016 to March 2017), Hospitals = 405	Patient-to-nurse ratio per shift (wards average at hospital level)	In-hospital mortality, readmission within 30 days, length of hospital stay	Logistic regression analysis (for in-hospital mortality and readmission within 30 days), regression analysis (for length of hospital stay) Conf.: patient-level variables, hospital-level variables	Higher number of patient-to-nurse ratio associated with extended length of hospital stay but not associated with other outcomes
Morioka et al. [19]	Retrospective observational study Data: DPC, reporting on medical functions of hospital beds data	Patients = 48,797 (65 years or older, underwent hip surgeries and discharged from April 2016 to March 2017), Acute care hospitals = 404	Patient-to-nurse ratio per shift (wards average at hospital level)	In-hospital mortality, in-hospital pneumonia, in-hospital fracture, length of hospital stay	Multilevel logistic regression analysis Conf.: patient-level variables, hospital-level variables	Higher number of patient-to-nurse ratio associated with extended length of hospital stay but not associated with other outcomes
Hirose et al. [16]	Retrospective observational study Data: DPC, the Annual Report for Functions of Medical Institution	Patients = 645687 (aged 20–99 years, underwent major cancer surgeries from July 2010 to March 2018), Hospitals = 787	Patient-to-nurse ratio per shift (wards average at hospital level)	30-day in-hospital mortality, failure to rescue, post-operative complications	Restricted cubic spline regression analyses Conf.: patient-level variables, hospital-level variables	Higher number of patient-to-nurse ratio was associated with post-operative complication (J-shaped association) but not associated with other outcomes

DPC: Japanese Diagnosis Procedure Combination inpatient database; Conf.: confounders.

**Table 2:** Summary of the evidence for the association between nurse staffing level and outcomes in Japanese hospitals.

		Patient-to-Nurse Ratio			Nursing Hours per Patient Day		Nurse-to-Bed Ratio	
		Patient-to-Nurse Ratio Requirement in the Fee Schedule	Patient-to-Nurse Ratio per Shift		Registered Nurse Hours per Patient Day	Associate Nurse Hours per Patient Day		
		Hospital Level	Average Wards at Hospital Level	Nurse at Individual Level	Ward Level	Ward Level	Hospital Level	Ward Level
Patient outcome	Readmission		N.S. [18]					
	Hospitalization		N.S.^§^ [21]					
	In-hospital mortality		N.S. [16,18,19,23]				N.S.^†^ [23]	
	Failure to rescue		N.S. [16]				△^†^ [23]	
	Length of hospital stay		▲ [18,19]					
	In-hospital fracture		N.S. [19]				△ [20]	
	In-hospital pneumonia		N.S. [19]					
	Post-operative complications		▲^‡^ [16]				N.S.^†^ [23]	
	Seclusion							▽ [15]
	Pressure ulcer				N.S. [17]	△ [17]		
	Physical restraint				N.S. [17]	N.S. [17]		▽ [15]
	Error and/or near miss			N.S.^§^ [11]				
	Patient satisfaction with nursing care	△^§^ [14]						
Nursing care quality	Nurse-reported quality of care	N.S.^§^ [14]		N.S. [13]				
	Ability to provide quality nursing care			N.S. [13]				
Nurse outcome	Work engagement	N.S. ^§^ [10]						
	Job satisfaction	△^§^ [12]						
	Stressor of work	N.S.^§^ [14]						
	Response to stress (mental, physical)	N.S.^§^ [14]						
	Intention to leave	N.S.^§^ [22]						
	Decision to leave	N.S.^§^ [22, 24]						
	Nurse-reported better working environment	△^§^ [14]						
	Ward morale			▲ [13]				

Highlights with orange indicate more staff favors, blue indicates fewer staff favors, and green indicates no significant association between nurse staffing and outcome. △: indicates more staff associated with lower risk/better outcome (*p* < 0.05). ▲: indicates fewer staff (higher nurse workload) associated with higher risk/worse outcome (*p* < 0.05). ▽: indicates more staff associated with higher risk/worse outcome (*p* < 0.05), N.S.: not statistically significant association. †: In this study, the combined physician-to-bed ratio (below or above median) and nurse-to-bed ratio (below or above median) variables were used as staffing variables. ‡: This study shows a reverse J-shaped association with post-operative complications with a threshold of patient-to-nurse ratio per shift of 5.4. §: This is a result from bivariate analysis.

## Data Availability

Not applicable.

## Supplementary Materials

The following supporting information can be downloaded at: https://www.mdpi.com/article/10.3390/healthcare10061052/s1, Table S1: Search term; Table S2: Detail characteristics of studies in the review; Table S3: Risk of bias assessment.
