# Supplementary material for: Evidence of the Association between Nurse Staffing Levels and Patient and Nurses’ Outcomes in Acute Care Hospitals across Japan: A Scoping Review
Source: Healthcare (Basel). 2022 Jun 6;10(6):1052. doi: 10.3390/healthcare10061052 (PMC9223072; doi:10.3390/healthcare10061052)
Supplement: Supplementary file 1 [file healthcare-10-01052-s001.zip › TableS1_searchtermEnglish_CINAHL.pdf]

Table S1. Search term

ICHUSHI, CiNii

	search term	further information
1	(kango-shi or kango-shoku or kango-shokuin or kango-staff) and (number or number of people or deployment)	Kango-shi, kango-shoku, kango-shokuin, and kango-staff are synonyms of "nurse" in Japanese
2	quantity of nursing	
3	quantity of nursing care or nursing care delivery system	
4	patient and (kango-shi or kango-shoku or kango-shokuin or kango-staff) and (hi or ritsu or hi-ritsu)	Hi, ritsu, and hi-ritsu are synonyms of "ratio" in Japanese
5	hospital bed and (kango-shi or kango-shoku or kango-shokuin or kango-staff) and (hi or ritsu or hi-ritsu)	
6	1 or 2 or 3 or 4 or 5	
7	patient and outcome	
8	quality of nursing or quality of nursing care	
9	(shoku-mu or gyoumu or shigoto) and satisfaction	Shoku-mu, gyoumu and shigoto are synonyms of "job" in Japanese.
10	burnout or moetsuki	Moetsuki is a synonym of "burnout" in Japanese.
11	risyoku or taisyoku or kyuusyoku or kyuuka or kekkin	Risyoku and taisyoku are synonyms of "turnover" in Japanese.<br>Kyuusyoku, kyuuka, and kekkin are synonyms of "leaving from work" in Japanese
12	7 or 8 or 9 or 10 or 11	
13	6 and 12	

Pubmed

1	nurse and (staffing or number or deployment or ratio or rate or proportion)
2	quantity of nursing care
3	nursing system
4	1 or 2 or 3
5	patient and outcome
6	quality of nursing care

7	job satisfaction
8	burnout
9	turnover or (sick leave) or leave
10	nurse sensitive outcome
11	nurse sensitive indicator
12	patient satisfaction
13	5 or 6 or 7 or 8 or 9 or 10 or 11 or 12
14	Japan and hospital
15	4 and 13 and 14

CINHAL

1	nurse and (staffing or number or deployment or ratio or rate or proportion)
2	quantity of nursing care
3	nursing system
4	1 or 2 or 3
5	patient and outcome
6	quality of nursing care
7	job satisfaction
8	burnout
9	turnover or (sick leave) or leave
10	nurse sensitive outcome
11	nurse sensitive indicator
12	patient satisfaction
13	5 or 6 or 7 or 8 or 9 or 10 or 11 or 12
14	(Japan or Japanese) and (hospital or acute care)
15	4 and 13 and 14
