# Supplementary material for: Evidence of the Association between Nurse Staffing Levels and Patient and Nurses’ Outcomes in Acute Care Hospitals across Japan: A Scoping Review
Source: Healthcare (Basel). 2022 Jun 6;10(6):1052. doi: 10.3390/healthcare10061052 (PMC9223072; doi:10.3390/healthcare10061052)
Supplement: Supplementary file 1 [file healthcare-10-01052-s001.zip › TableS2_risk of bias assessment.pdf]

Table S2. Risk of bias assessment

Author(s), year	Confounding	Participants	interventions	Deviations	Missing data	Outcomes	Results	Overall bias
Anzai et al., 2014	Low	Low	Serious	Low	NI	Serious	Moderate	Serious
Fujimura et al., 2011	Critical	NI	Low	Critical	NI	Serious	Serious	Critical
Fukasawa et al., 2018	Moderate	Serious	Moderate	Low	NI	Moderate	Low	Moderate
Hirose et al., 2021	Low	Low	Moderate	Serious	NI	Low	Low	Moderate
Ibe et al., 2008	Moderate	Low	Low	Low	NI	Moderate	Low	Moderate
Ito et al., 2018	Critical	Low	Low	Critical	NI	Serious	Serious	Critical
Kaneko et al., 2008	Critical	Low	Serious	Low	NI	Critical	Critical	Critical
Morioka et al., 2020	Low	Low	Moderate	Serious	NI	Low	Low	Moderate
Morioka et al., 2021	Low	Low	Moderate	Serious	NI	Low	Low	Moderate
Morita et al., 2017	Low	Low	Moderate	Serious	NI	Low	Low	Moderate
Nawata et al., 2006	Critical	Low	Low	Low	Serious	Low	NI	Critical
Namba et al., 2014	Critical	Low	Low	Critical	NI	Serious	Serious	Critical
Suzuki et al., 2006	Critical	Low	Low	Critical	NI	Low	Serious	Critical
Tei-Tominaga, 2013	Serious	Low	Low	Critical	NI	Serious	Moderate	Serious
Yasunaga et al., 2012	Low	Low	Moderate	Serious	NI	Low	Serious	Serious

NI: No Information
