# Supplementary material for: Evidence of the Association between Nurse Staffing Levels and Patient and Nurses’ Outcomes in Acute Care Hospitals across Japan: A Scoping Review
Source: Healthcare (Basel). 2022 Jun 6;10(6):1052. doi: 10.3390/healthcare10061052 (PMC9223072; doi:10.3390/healthcare10061052)
Supplement: Supplementary file 1 [file healthcare-10-01052-s001.zip › TableS3_Characteristics of studies in the review detail ver.pdf]

Table S3. Detail characteristics of studies in the review

Author, year	Design & data source	Setting & participants & sample size	Measurement of staffing	Outcome measures	Potential confounders measured and included in analysis	Results
Nawata *et al*., 2006	Two-wave questionnaire survey<br><br>1st survey: October 1993<br>2nd survey: October 1999	Psychiatric hospitals = 18; Patients (in 1st survey) = 2386; Patients (after 2nd survey) = 1131	**Number of patients per nurse** at the 1st survey: 4 categories (1 nurse or aide per 2.5-3 patients, 1 nurse or aide per 4 patients, 1 nurse or aide per 5-6 patients, or lower than 1 nurse or aide per 5-6 patients)	**Percentage of followed-up period hospitalized:** cumulative number of days hospitalized from 1st survey to 2nd survey/2192 days as number of days from first survey to 2nd survey	Not applicable (multivariate analysis was not conducted)	Bivariate analysis (no detailed information)<br>correlation coefficient = .102 (between the staffing level and the percentage of followed-up period hospitalized)
Suzuki *et al*., 2006	Longitudinal questionnaire survey from June 2003 to December 2003	Novice full-time nurses in 2003 = 1,203<br>University hospitals = 20	**Number of patients per nurse:** 2 categories **(**2.5:1 or 2:1)	**Rapid turnover among novice nurses** confirmed by information from the director of the nursing service department in each hospital	Not applicable (multivariate analysis was not conducted)	Bivariate analysis<br>no statistically significant difference between nurses who turned over and those who did not
Ibe *et al*., 2008	Longitudinal questionnaire survey from 1 November 2005 to 31 January 2006	Hospital nursing unit managers = 98<br>Hospitals = 42 (hospitals who attended the Research Committee of the Nurse Staffing System for Hospital Safety in 2003)	**Nurse hours per patient (daily):** the productive hours worked by nursing staff (registered nurses, associate nurses, and care workers) divided by the average number of inpatients	**Pressure ulcer:** all patients on the study day with stage I, II, III, or IV ulcers divided by the average daily number of patients (16 years old and older)<br><br>**Physical restraint:** all patients on the study day with restraints (limb, vest, side rails, other) divided by the average daily number of patients (16 years old and older)	Associate nurse hours per patient (daily), other hours per patient (daily), registered nurses hours, Nursing- care Needs Score	**Pressure ulcer:**<br>multiple regression analysis<br>standardized coefficient of registered nurse hours per patient (daily) = 0.321 ($p = .072$)<br><br>**Physical restraint:**<br>multiple regression analysis standardized coefficient of registered nurse hours per patient (daily) = 0.156 ($p = .353$)

Kaneko *et al.*, 2008	Cross-sectional questionnaire survey during November and December 2005	Nurses working in wards = 787 (candidates = 1,339)<br>Hospitals = 6 (clinical training hospital and emergency designated hospitals in a designated city)	**Number of patients per nurse:** self-reported by respondents	**Medical error and mistakes:** the experience of medical error and mistakes during 6 months before questionnaire survey	age	Univariable logistic regression<br>an odds ratio of number of patients per nurse in daytime shift = 1.10 [95% CI, 0.97-1.06]<br>an odds ratio of number of patients per nurse in nighttime shift = 0.99 [95% CI, 0.95-1.03]
Fujimura *et al.*, 2011 Inpatient survey	Questionnaire survey from January 2008 to December 2008	Inpatients = 202 (inpatients who were discharged from the Departments of Cardiology and Metabolism of an advanced treatment hospital)	**Number of patients to nurse ratio:** 2 categories **(**7:1 or 10:1)	**Quality of life**: The Japanese version of the Short-Form 36-Item Health Survey (SF-36)<br><br>**Inpatient satisfaction**: 7 items (Explanation, Easiness of Consultation, Dealing with Patients, Reliability, Coping with Patients' Pain/Indisposition, Coping with Patients' Anxiety/Mental Care, and Satisfaction) from a nationwide survey in 2003 (partially modified)	Not applicable (multivariate analysis was not conducted)	**Quality of life:**<br>bivariate analysis<br>no statistically significant difference in each norm of SF-36 (Table 1)<br><br>**Inpatient satisfaction**:<br>bivariate analysis<br>the 7:1 system showed statistically greater satisfaction in all subcategories than the 10:1 system (Table 1).
Fujimura *et al.*, 2011 Medical workers survey	Questionnaire surveys in February 2008 (under the 10:1 system) and in October 2008 (after the introduction of the 7:1 system)	1st survey<br>Physicians = 36<br>Nurses = 14<br><br>2nd survey<br>Physicians = 33<br>Nurses = 25	**Number of patients to nurse ratio:** 2 categories **(**7:1 or 10:1)	**Job satisfaction**: the physicians' and nurses' estimation of their Working Hours, Income, Number of Physicians in the Ward, and Number of Nurses in the Ward<br><br>**Effects of the diagnosis procedure combination (DPC)**: DPC-specific questionnaire consisting of 11 items	Not applicable (multivariate analysis was not conducted)	Results of nurses alone (did not extract results of physicians)<br><br>**Job satisfaction:**<br>bivariate analysis<br>Nurses under the 7:1 system judged that their working hours were shortened compared to those under the 10:1 system (their real working hours were unaltered).

				**Stress of medical workers**: the Simplified Job Stressor Questionnaire (Workload, Control, Personal Relations, Conformity, and Support by Coworkers/Supervisor and responses to stress with Mental Stress and Physical Stress)		Nurses under the 7:1 system judged that number of nurses in the ward were significantly higher than those under the 10:1 system.<br><br>**Effects of the diagnosis procedure combination (DPC)**:<br>bivariate analysis<br>Improvement of working environment rated by nurses under the 7:1 system was higher (p < .05) than that those under the 10:1 system.<br><br>**Stress of medical workers:**<br>bivariate analysis<br>Workload +; 13 (92.9%) at 1st stage v 24 (96.0%) at 2nd stage<br>Control +; 5 (35.7%) v 10 (40.0%)<br>Personal relations +; 1 (7.1%) v 4 (16.0%)<br>Conformity +; 0 (0.0%) v 2 (8.0%)<br>Support by coworkers/superior +; 0 (0.0%) v 1 (4.0%)
Yasunaga *et al*., 2012	Retrospective observational study<br><br>Data: DPC database	Patients = 131,394 (underwent elective cancer surgery*[1] between 2007 and 2008)	**Number of physicians per 100 beds and the number of nurses per 100 beds:** 4 categories Group A (below median PBR and below median NBR), Group B (below median PBR and	**Postoperative complications:** surgical site infection, peritonitis, sepsis, respiratory complications (pneumonia, postprocedural respiratory disorders or respiratory failure), pulmonary embolism, cardiac events (acute coronary events or heart failure),	age, sex, Charlson comorbidity index, hospital volume (low, medium, high)<br><br>A reference in staffing level = Group A	**Failure to rescue**:<br>logistic regression analysis<br>an odds ratio between Groups A and D = 0.76 [95%CI, 0.63-0.90], p = .002<br>an odds ratio between Groups A and B = 0.94 [0.78-1.13], p = .505

	the Survey of Medical Institutions data		above median NBR), Group C (above median PBR and below median NBR), and (iv) Group D (above median PBR and above median NBR).<br><br>The number of nurses = the full-time equivalent numbers of licensed nurses	stroke (cerebral infarction or hemorrhage), and acute renal failure<br><br>**In-hospital mortality**<br><br>**Failure to rescue:**<br>the proportion of in-hospital death cases among those who had experienced a postoperative complication		an odds ratio between Groups A and C = 0.91 [0.73-1.13], p = .379<br><br>**Postoperative complications**:<br>logistic regression analysis<br>an odds ratio between Groups A and D = 1.01 [95%CI, 0.90-1.13], p = .918<br><br>**In-hospital mortality**:<br>logistic regression analysis<br>an odds ratio between Groups A and D = 0.82 [0.71-0.95], p = .009
Namba *et al*., 2013	Cross-sectional questionnaire survey from April 22 to May 28 2009	Full-time nurses of = 919 (candidates = 2213)<br>Hospitals = 15 (97 wards) in a prefecture	**Number of patients to nurse ratio:** 2 categories **(**7:1 or 10:1)	**Measurement of Nurses' Job Satisfaction:** total score and sub-scale scores (pay, professional status, doctor-nurse relationship, administration, autonomy, task requirement, interaction)<br><br>**Retention Potential Scale for Nurses:** total score and sub-scale (organizational satisfaction, sense of belonging to organization, previous retention degree, intention to continue working）	Not applicable (multivariate analysis was not conducted)	**Measurement of Nurses' Job Satisfaction**:<br>bivariate analysis<br>mean (±SD) sub-scale score of professional status = 3.64 ± 0.76 (7:1) and 3.52 ± 0.83 (10:1), p = .008,<br>mean (±SD) sub-scale score of interaction = 3.93 ± 0.83 (7:1), 3.72±0.88 (10:1), p = .001,<br>other sub-scale scores p > .05<br><br>**Retention Potential Scale for Nurses**:<br>bivariate analysis<br>total score of 3 groups (stable to retent, unstable to retent, unable to retent), p > .05<br>organizational satisfaction, p = .000<br>other sub-scale scores, p > .05

					Employment and organizational characteristics: desirable position (in a word), working more than 51 hours per week, break facilities and amount of permitted rest time in the hospital, hospital type (public hospital or not),<br><br>Individual factors: job readiness (being personally suited for nursing work), 12-item General Health Questionnaire, 13-item Cumulative Fatigue, psychosocial factors in the work environment: 2 subscales of social support in 57-item simplified work-related stress questionnaire (supervisor support, coworker support), Japanese short version of the Copenhagen Psychosocial Questionnaire (quantitative demands, cognitive demands, emotional demands, demands for hiding emotions, sensory demand), presence of a role model	
Tei-Tominaga, 2013	Cross-sectional questionnaire survey in December 2009	Newly graduated nurses =493 (candidates = 1,477)<br>Hospitals =353	**Number of patients to nurse ratio:** 2 categories **(**7:1 or 10:1)	**Intention to leave**: 6-item scale developed by Tei and Yamazaki (2003) dichotomized by the upper quartile<br><br>**Decision to resign**: dichotomous data using one item, ‘‘Will you resign from your current organization within this fiscal year (within 3 months)?		**Intention to leave**:<br>bivariate analysis<br>p = .043<br>multivariate logistic regression analysis<br>p = .291<br><br>**Decision to resign**:<br>bivariate analysis<br>p = .002<br>multivariate logistic regression analysis<br>p = .060
Anzai *et al*., 2014	Cross-sectional questionnaire survey	Nurses = 223 (candidates = 341)<br>Acute-care inpatient wards = 12<br>Hospital = 1	**Number of patients in usual day shift**:<br>self-rated by 4-point scale (<5, 5, 6, ≧7)	**Ability to provide quality nursing care**: 4-point Likert scale from strongly disagree to strongly agree<br><br>**Quality of care**: 11-point scale (0 = poor, 10 = excellent).	Demographic characteristics: gender, nursing experience, nursing education.<br><br>Work characteristics: nurse manager position, shift type, number of total shifts, percentage of day shifts, hours overtime during previous work.	**Ability to provide quality nursing care**:<br>multiple regression analyses<br>β = 0.02, p > .05<br><br>**Quality of patient care**:<br>multiple regression analyses<br>β = -0.05, p > .05

				**Ward morale**: 11-point scale (0 = very low, 10 = very high).	Japanese version of Practice Environment Scale of the Nursing Work Index (PES-NWI) subscales: nurse participation in hospital affairs, nursing foundations for quality of care, nurse manager ability, leadership, and support of nurses, staffing and resource adequacy, collegial nurse-physician relations.	**Ward morale**:<br>multiple regression analyses<br>β = -0.07, p > .05
Morita *et al*., 2017	Retrospective cohort study<br><br>Data:<br>DPC, the Surveys for Medical Institution	Patients = 770,373<br>(50 years or older and underwent planned major surgery*[2] for some forms of cancer or cardiovascular diseases from July 2010 to March 2014)<br>Hospitals = 1,074	**Nurses-to-occupied bed ratio:** The number of inpatient nurses per 100 occupied beds for each hospital. The number of nurses included the full-time equivalent registered nurses and licensed practical nurses at inpatient hospital wards and units, but it did not include nursing assistants. NBR was categorized into quartiles (lowest, lower middle, higher middle, and highest), with an approximately equal number of patients in each group.	**The occurrence of in-hospital bone fractures:**<br>identified by ICD-10 codes (in parentheses) and postoperative procedure codes associated with fractures	Patient age, sex, smoking status, body mass index, ADL score for walking on a flat floor, Charlson comorbidity index, comorbid medical conditions related to falls or bone fracture, and use of drugs	**In-hospital bone fractures**:<br>logistic regression analysis<br>adjusted odd ratio (the highest nurses-to-occupied bed ratio v the lowest nurses-to-occupied bed ratio) = 0.67 [95 % CI, 0.44–0.99], p = .048

Fukasawa *et al.*, 2018	Secondary analysis of clinical database from April 2015 to March 2017	Admissions = 10,013<br>Hospitals = 23 (participating in the Psychiatric Electronic Clinical Observation (PECO) system)	**Number of nurses per bed**: Nurse per 10 beds in each ward (ward level)	**Use of seclusion or mechanical restraint during the first 90 days of admission**: the number of admissions exposed to at least one episode of seclusion or mechanical restraint	Sex, age, psychiatric diagnosis (ICD10), Admission form at the time of admission (voluntary / involuntary), dose of antipsychotics, Global Assessment of Functioning (GAF), type of ward (ordinary / acute), location (rural / urban), random parameter	**Seclusion:**<br>multilevel logistic regression analysis<br>adjusted odds ratio = 2.36 [95% CI 1.55-3.60]<br><br>**Mechanical restraint:**<br>multilevel logistic regression analysis<br>adjusted odds ratio = 1.74 [95% CI, 1.35-2.24]
Ito *et al*., 2018	Cross-sectional questionnaire survey from 5 December 2013 to 25 December 2013	Shift-work nurses = 1,275 (candidates = 1,800)<br>Hospitals = 13 (randomly selected from 111 hospitals of the same organization which are not established by municipalities but are expected to play public roles)	**Number of patients per nurse**: 5 categories (2 to 1 or 4 to 1, 7 to 1, 10 to 1, 13 to 1 or 15 to 1)	**Work engagement:** Japanese version of the Utrecht Work Engagement Scale	Not applicable (multivariate analysis was not conducted)	Bivariate analysis (no detailed information)<br>no statistically significant relationship between patients-to-nurse ratio and scores of work engagement
Morioka *et al*., 2020	Retrospective observational study<br><br>Data:<br>DPC, reporting on medical functions of	Patients = 20,393 (dementia, 65 years or older, underwent hip surgeries*[3] and discharged from April 2016 to March 2017)<br>Hospitals = 405	**Patient-to-nurse ratio:**<br>an average inpatient-to-nurse ratio per shift<br><br>Nurses = national licensed nurse and associate nurse licensed by prefectural government	**In-hospital mortality:**<br>the all-cause death during hospitalization<br><br>**Readmission within 30 days:**<br>readmission to the same hospital within 30 days post-discharge<br><br>**Length of hospital stay:**	**In-hospital mortality:**<br>dementia care status, nurse staffing, skill mix, sex, body mass index, Charlson comorbidity index, type of surgery, psychotropic drug use, and number of hospital beds<br><br>**Readmission within 30 days:**	**In-hospital mortality:**<br>logistic regression analysis<br>an adjusted odds ratio of patient-to-nurse ratio = 1.03 [95% CI, 0.90-1.17], p = .715<br><br>**Readmission within 30 days**:<br>logistic regression analysis

	hospital beds data			the number of hospitalization days from admission to discharge.<br><br>The patients who died during hospitalization were excluded in the calculation of readmission and length of hospital stay.	dementia care status, nurse staffing, skill mix, sex, body mass index, Charlson comorbidity index, type of surgery, psychotropic drug use, and types of residence after discharge<br><br>**Length of hospital stay:**<br>dementia care status, nurse staffing, skill mix, sex, body mass index, Charlson comorbidity index, type of surgery, psychotropic drug use, types of residence before admission and after discharge, interaction term between types of residence before admission and after discharge, and number of hospitals	an adjusted odds ratio of patient-to-nurse ratio = 1.09 [95% CI, 0.98-1.22], p = .125<br><br>**Length of hospital stay**:<br>regression analysis<br>an adjusted coefficient of patient-to-nurse ratio = 2.25 [95% CI, 1.00-3.51], p < .001
Morioka *et al*., 2021	Retrospective observational study<br><br>Data:<br>DPC, reporting on medical functions of hospital beds data	Patients = 48,797 (65 years or older, underwent hip surgeries*[4] and discharged from April 2016 to March 2017)<br>Acute care hospitals = 404	**Patient-to-nurse ratio:**<br>The average number of in-patients per nurse and associate nurses per shift at general acute care wards<br><br>The average number of in-patients per nurse per shift = (the number of in-patients in the general care wards during the past year * 3 *8) / (the number of full-time equivalent nurses working at general wards/1800)	**In-hospital mortality:**<br>all-cause death during hospitalization<br><br>**In-hospital pneumonia:**<br>identified by the type of pneumonia (community-acquired, in-hospital, and other)<br><br>**In-hospital fracture:**<br>at least one fracture post-admission<br><br>**Length of hospital stay:**<br>the number of hospitalization days from admission to discharge	**Length of hospital stay:**<br>sex, body mass index, Charlson comorbidity index, type of surgery, psychotropic drug use, types of established organization of hospitals and number of hospital beds	**In-hospital mortality:**<br>multilevel logistic regression analysis<br>an odds ratio of patient-to-nurse ratio = 1.07 [95% CI = 0.96–1.19], p = .253<br><br>**In-hospital pneumonia**:<br>multilevel logistic regression analysis<br>an odds ratio of patient-to-nurse ratio = 0.99 [95% CI = 0.64-1.52], p = .962<br><br>**In-hospital Fracture**:<br>multilevel logistic regression analysis<br>an odds ratio of patient-to-nurse ratio = 1.08 [95% CI = 0.86-1.35], p = .524

						**Length of hospital stay**:<br>multilevel logistic regression analysis<br>positive association between patient-to-nurse ratio and length of hospital stay with 7.8% [95% CI = 4.2-11.5%], p < .001
Hirose *et al*., 2021	Retrospective observational study<br><br>Data:<br>DPC, the Annual Report for Functions of Medical Institution in 2014	Patients = 645687 (aged 20-99 years, underwent major cancer surgeries*[5] from July 2010 to March 2018)<br>Hospitals = 787	**Patient-to-nurse ratio per shift**:<br>total inpatient days / number of nursing staff × 1800 hours / 24 hours<br><br>Nurse: registered nurses who worked in general wards of each hospital	**30-day in-hospital mortality**<br><br>**Failure to rescue:** the number of six treatable postoperative complications (deep venous thrombosis, pneumonia, cardiac arrest, shock, gastrointestinal bleeding and sepsis) / the number of death of patients suffering from the complications<br><br>**Postoperative complications:**<br>deep venous thrombosis, pneumonia, cardiac arrest, shock, gastrointestinal bleeding and sepsis	Patient-level variables:<br>age, sex, smoking status, activities of daily living, Charlson comorbidity index, body mass index, type of surgery, laparoscopic surgery, cancer recurrence, and chemotherapy use<br><br>Hospital-level variables:<br>type of hospital, hospital volume, patient turnover rate and number of physicians per 100 occupied beds	**Failure to rescue and 30-day in-hospital mortality:**<br>restricted cubic spline regression analyses<br>insignificant associations of patient-to-nurse ratio with no threshold<br><br>**Postoperative complications:**<br>restricted cubic spline regression analyses<br>a reverse J-shaped association with postoperative complications with a threshold of patient-to-nurse ratio per shift of 5.4

footnotes: DPC, Japanese Diagnosis Procedure Combination inpatient database; CI, confidence interval; SD, standard deviation

*1 Elective cancer surgeries included lung lobectomy for lung cancer (excluding pneumonectomy), esophagectomy for esophageal cancer, gastrectomy for gastric cancer, colorectal cancer surgery (including colectomy for colon cancer and anterior resection or abdominoperineal resection for rectal cancer), hepatectomy for hepatic cancer, and pancreatectomy for pancreatic cancer.

*2 Planned major surgeries included surgeries for intracranial tumor, pharyngeal cancer, breast cancer, respiratory or mediastinal cancer, aerodigestive cancer, genitourinary cancer, and cardiovascular diseases.

*3 Hip surgeries included osteosynthesis, bipolar hip arthroplasty, and total hip arthroplasty.

*4 Hip surgeries included osteosynthesis, bipolar hip arthroplasty, and total hip arthroplasty.

*5 Major cancer surgeries included esophagostomy, gastrectomy, colorectal resection, hepatectomy, pancreatectomy, and pulmonary lobectomy.
